# Stepwise substrate translocation mechanism revealed by free energy calculations of doxorubicin in the multidrug transporter AcrB

**DOI:** 10.1038/srep13905

**Published:** 2015-09-14

**Authors:** Zhicheng Zuo, Beibei Wang, Jingwei Weng, Wenning Wang

**Affiliations:** 1 Shanghai Key Laboratory of Molecular Catalysis and Innovative Materials and Collaborative Innovation Center of Chemistry for Life Sciences, Department of Chemistry; 2 Institutes of Biomedical Sciences, Fudan University, Shanghai, P.R. China

## Abstract

AcrB is the inner membrane transporter of the tripartite multidrug efflux pump AcrAB-TolC in *E. coli*, which poses a major obstacle to the treatment of bacterial infections. X-ray structures have identified two types of substrate-binding pockets in the porter domains of AcrB trimer: the proximal binding pocket (PBP) and the distal binding pocket (DBP), and suggest a functional rotating mechanism in which each protomer cycles consecutively through three distinct conformational states (access, binding and extrusion). However, the details of substrate binding and translocation between the binding pockets remain elusive. In this work, we performed atomic simulations to obtain the free energy profile of the translocation of an antibiotic drug doxorubicin (DOX) inside AcrB. Our simulation indicates that DOX binds at the PBP and DBP with comparable affinities in the binding state protomer, and overcomes a 3 kcal/mol energy barrier to transit between them. Obvious conformational changes including closing of the PC1/PC2 cleft and shrinking of the DBP were observed upon DOX binding in the PBP, resulting in an intermediate state between the access and binding states. Taken together, the simulation results reveal a detailed stepwise substrate binding and translocation process in the framework of functional rotating mechanism.

Multidrug resistance (MDR) efflux pump confers resistance against a wide range of structurally or chemically unrelated antibiotics, and represents a serious impediment to improved healthcare^1^. MDR efflux pumps are membrane proteins that actively expel toxins out of the cell. One of the most studied MDR systems is the tripartite AcrAB-TolC pump in *Escherichia coli*, which is constituted of an inner membrane transporter AcrB belonging to the resistance-nodulation-division (RND) superfamily, a channel-tunnel outer membrane protein TolC, and a periplasmic adaptor protein AcrA. AcrB acts as the dynamo of the tripartite system by harnessing proton motive energy across the inner membrane to collect and extrude substrate toward TolC^34^.

Structural and biochemical studies have demonstrated that AcrB forms a homotrimer^2^,^3^,^4^,^5^,^6^,^7^,^8^,^9^,^10^,^11^, with each protomer composed of a transmembrane (TM) domain, a porter domain and a TolC-docking domain (Fig. 1a). Each TM domain and porter domain embeds a proton-relay pathway and a substrate translocation pathway, respectively, whereas three TolC-docking domains enclose a central funnel for substrate to move onward to the TolC lumen^12^. Although early crystallographic studies obtained a fully symmetric structure of AcrB trimer at 3.5 Å, subsequently solved asymmetric crystal structures at 1.9 ~ 3.5 Å demonstrated that the three protomers adopt distinct conformations dubbed as access (or loose), binding (or tight), and extrusion (or open) states (Fig. 1b)^2^,^3^,^4^,^5^,^6^,^7^,^8^,^9^,^10^,^11^. Based on the asymmetrical crystal structures, a functional rotating mechanism has been proposed that each protomer cycles consecutively through the three conformational states during the efflux of substrate^4^,^7^,^8^. The inter-state transitions bring evident changes to the TM domain, especially to the proton-relay network at the core of TM4 and TM10 helices, varying the protonation states of titratable residues and the degree of solvent exposure^13^. The transitions also entail variations in the porter domain, especially in two binding pockets along the substrate translocation pathway (Fig. 1b). The distal (or deep) binding pocket (DBP) lying between the subdomains PC1 and PN2 (Fig. 1b) is rich in phenylalanine residues^3^,^4^,^6^,^14^,^15^,^16^, whereas the proximal binding pocket (PBP, also called access binding pocket) involving more hydrophilic residues is located in the cleft between PC1 and PC2 subdomains (Fig. 1b)^2^,^3^,^6^,^9^,^17^, segregated from the DBP by a flexible switch-loop (or G-loop, Phe617 loop) of ~11 residues (Fig. 1a,b)^3^,^14^,^18^,^19^. The substrate accessibility and affinity of the binding pockets are evidently affected by the inter-state transitions of protomer. Structural information suggests that the PBP of the access protomer is accessible to high-molecular-mass substrates^2^,^3^,^6^, and the DBP will come into operation after the access→binding transition. For substrates with lower molecular mass, however, the DBP of binding protomer is directly open to them^6^. During the subsequent binding→extrusion transition, the DBP shrinks and expels the substrate to the central funnel^4^,^17^,^20^,^21^.

Despite the accumulating experimental data supporting the functional rotating mechanism, many detailed aspects of substrate binding and translocation in the two binding pockets remain elusive. Among these are the conformational changes of the protein required during substrate translocation and the energy barrier caused by the switch-loop as indicated by a number of biochemical studies^3^,^14^,^18^,^19^. To elucidate these questions, it is necessary to provide a quantitative description of the energetics of substrate transportation through the translocation pathway^22^,^23^,^24^. Computational simulation has been proved to be a powerful tool in studying the dynamics of AcrB^25^,^26^, the substrate-protein interaction^15^,^16^,^27^ and the translocation motion of substrate inside the transporter^20^,^21^,^23^,^24^,^28^. In this contribution, we performed over 1 μs atomic simulations with explicit solvent and lipid membrane to calculate the potential of mean force (PMF) of the translocation of the antibiotic drug doxorubicin (DOX) (Fig. 1c) inside the binding protomer of AcrB. The adaptive biasing force (ABF) method was used for the PMF calculation, which integrates the novel ideas of unconstrained thermodynamic integration and adaptive bias based on a local, running estimate of the free energy derivative, and has been successfully applied to many problems in chemistry and biology^29^,^30^. Here the reaction coordinate (RC) along the putative vestibule path (Fig. 1a)^6^,^7^,^8^,^9^,^24^,^31^ was defined for the free energy calculations. The derived PMF profile clearly features two deep energy basins, corresponding to the sites at the PBP and DBP, respectively. Obvious conformational changes of AcrB during substrate translocation were observed, including the PC1/PC2 cleft closing and the DBP shrinking upon DOX binding at the PBP of binding protomer, giving rise to a new intermediate state. The biological implications of these observations were discussed.

## Results

The calculated PMF profile is shown in Fig. 2a, the zero point of which is set at the DBP. The convergence of the ABF simulations was examined in several aspects. First, the final PMF profile is barely influenced by further extension of the simulation time by 10 ns in each window (Figure S1a, Supporting information), and the estimated standard errors are all below 1.0 kcal/mol (Fig. 2a). On the other hand, the average forces, i.e. the first derivative of free energy are continuous (Figure S1b, Supporting information). These analyses demonstrate that the simulation is well converged.

### Temporary binding at the PC-loop near the vestibule entrance

Along the RC, the PMF profile shows a shallow energy minimum at 13.6 Å (Fig. 2a), near the vestibule entrance. Its energy is ~1.5 kcal/mol lower relative to the neighboring areas, indicating temporary binding of DOX at the site during the translocation process. This local minimum is mainly resulted from the interactions of DOX with residues Glu673 and Thr678 on the loop (residues 669–679) connecting PC1 and PC2 subdomains (designated as PC-loop hereafter), Glu866 at the N-terminal end of TM8 and Phe563 on the loop connecting TM7 and PC1 (Fig. 2b). In the asymmetric cocrystal structure of AcrB/minocycline (PDB ID: 4DX5)^3^, two detergent molecules (dodecyl-β-D-maltoside and dodecyl-α-D-maltoside) were found to bind at the TM8/TM9 groove and the lateral PC1/PC2 cleft (i.e. PBP) of binding protomer, respectively. The weak DOX binding site at RC = 13.6 Å is approximately at the midpoint between the locations of two detergent molecules (Figure S2a, Supporting information). Interestingly, the helical structure adopted by the PC-loop (Fig. 2b) was dominant only when DOX resided around the weak binding site at RC = 13.6 Å. Turn or coil structures were more frequently observed when DOX was far from the site (Figure S2b, Supporting Information). This implies that the PC-loop undergoes conformational transitions upon binding of substrate, though the functional meaning of this change is not clear.

### DOX stably binds to the DBP

More inside the translocation pathway, the free energy profile is featured by two deep energy basins (Fig. 2a). The one centered at ~40 Å corresponds to DOX binding at the DBP, which has been clearly identified in many AcrB X-ray structures^3^,^4^,^6^,^11^. The relative free energy inside the DBP is ~−6 kcal/mol with respect to the entrance region (Fig. 2a). Previous coarse-grained model based calculations estimated the corresponding free energy of ~−3.6 kcal/mol when taking the “hydrophobicity” parameter 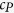 as 0.2^24^ (the 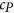 value of DOX is near 0.29 as calculated from the data in DrugBank^32^). In two AcrB/DOX co-crystal structures (PDB IDs: 2DR6^4^ and 4DX7^3^), DOX shows two different orientations inside the DBP. Our simulation demonstrates a highly populated orientation different from either of them (Figure S3, Supporting Information), further indicating the variety of DOX binding mode inside the DBP. The residues involved in binding DOX shown by the simulation are generally consistent with the crystal structures and biochemical experiments (Table S1, Supporting Information). The daunosamine moiety of DOX forms hydrogen interactions with Gln89 on PN1, whereas the aglycone moiety immerses in the phenylalanine-rich groove of DBP, sharing extensive van der Waals interactions with Phe178 and Ile277 on PN2, Ile626 and Phe628 on PC1, and Phe615 and Phe617 on the switch-loop (Fig. 2c; Table S1, Supporting Information). Most of these residues (Gln89, Phe178, Ile277, Phe628 and Phe617) are verified by fluorescence assays as they are accessible to AcrB substrate Bodipy-FL-maleimide and/or CPM/pyrene-maleimide^12^.

### DOX binds to the PBP with a comparable affinity as that of the DBP

The other deep basin on the PMF profile centered at 27.8 Å corresponds to DOX binding at the PBP of binding protomer (Fig. 2a). The optimal binding site of DOX is at the innermost region of the PBP, much closer to the switch-loop (Fig. 2d) than the DOX dimer (PDB ID: 4DX7) or other molecules in the access protomer observed in crystal structures^2^,^3^,^6^,^9^. The energy depth of this binding site is comparable to that of the DBP, slightly lower by 0.1 kcal/mol (Fig. 2a). This is surprising since the PBP of binding protomer was previously considered as a low affinity site for AcrB substrates^3^, especially for small substrates like DOX^6^. Inspecting the atomistic details of the interactions between DOX and the protein, however, rationalizes the sources of high binding affinity (Fig. 2e; Table S2, Supporting Information). The positively charged daunosamine moiety of DOX makes strong electrostatic interactions with the acidic residue cluster consisting of Asp681, Glu826 and Glu683 on PC2 (Fig. 2e). In the AcrB/DOX crystal structure (PDB ID: 4DX7)^3^, one of the two DOX molecules bound at the PBP forms analogous ionic interactions with the acidic cluster on PC2 (Table S2, Supporting Information). The aglycone moiety of DOX is mainly stabilized by van der Waals interactions with Met575, Phe664, Phe666 and Leu668 on PC1, and Phe617 on the switch-loop in the ABF simulations (Fig. 2e). These residues also interact with the aglycone moiety of the other DOX in the crystal structure^3^ (Table S2, Supporting Information). Therefore, our simulation shows that the one DOX molecule in the PBP of binding protomer can adjust to achieve an optimal binding by mimicking the interactions between the DOX dimer and the residues inside the PBP as observed in the crystal structure^3^. The interactions between DOX and AcrB are also validated by biochemical experiments. For instance, single site mutation such as F666W significantly reduces the efflux activity of DOX and erythromycin^6^. Several residues including Phe664, Phe666, Phe668 and Leu828 were also shown to be closely related to substrate transport^12^. Interestingly, in the AcrB/minocycline co-crystal structure (PDB ID: 4DX5)^3^, a detergent molecule dodecyl-α-D-maltoside was found to bind at the PBP of binding protomer. Though the binding was puzzling at that time, our simulation provides an explanation that the high ligand-binding affinity might be a general feature of the PBP in binding protomer, not only for DOX but also for other molecules.

### Conformational variations upon the binding at PBP

Along with the DOX binding at the PBP, significant conformational changes of the translocation pathway have been identified. The most prominent conformational change is the relative motion between subdomains PC1 and PC2. The PC1/PC2 cleft closed remarkably with the average distance between the centers of mass of PC1 and PC2 reduced by ~4 Å with respect to the crystal structure (Fig. 3a), which occludes the translocation tunnel toward the PC1/PC2 cleft entrance. To validate this conformational change, we performed two 50-ns unbiased MD simulations with DOX initially bound inside the PBP or DBP, respectively. In the PBP-bound system, the simulation showed a similar DOX orientation in the PBP as that in the ABF trajectory. At the same time, the closing motion of the PC1/PC2 cleft was observed, with the separation between PC1 and PC2 decreasing from 31 to 27.5 Å in 50 ns (Fig. 3b). In contrast, the PC1-PC2 distance fluctuated around 30 Å in the DBP-bound system, and seldom fell below 29 Å (Fig. 3b). Thus, the unbiased simulations are well consistent with the ABF simulations by showing that DOX binding at the PBP of binding protomer induces the closure of PC1/PC2 cleft. In the previous MD simulation studies of AcrB, the PC1/PC2 cleft motions were also observed although in the absence of any substrate^26^. These opening and closing motions indicate the intrinsic conformational flexibility of the cleft, whereas our simulations further demonstrate that DOX binding can regulate the conformation of the PC1/PC2 cleft by stabilizing it in either open or closed state depending on the location of DOX. In the crystal structures of AcrB with substrates bound at the PBP of access protomer, similar closing motion of the PC1/PC2 cleft, however, was not observed^2^,^3^,^6^,^9^. This may be attributed to the different state of protomer or to the differences in the residues involved in substrate binding. The substrates often contact with more lateral region of the PC1/PC2 cleft in crystal structures, but DOX gets into the innermost part of the cleft in our simulation (Fig. 2d). The closure of the PC1/PC2 cleft as DOX resides in PBP is reminiscent of the X-ray structures of the Zn(II)/proton antiporter ZneA, a member of the heavy metal efflux subfamily of RND pumps, in which the periplasmic cleft closes in the presence of Zn^2+^ at the proximal site (equivalent to the PBP in AcrB)^33^. In analogy with ZneA, the closure of the PC1/PC2 cleft in AcrB may also be important for preventing the backflow of substrate during translocation.

Another notable feature of the conformational rearrangements upon DOX binding in PBP is the shrinking of the DBP. The radius of gyration of DBP decreases evidently as DOX resides in the range between 26 Å to 28 Å along the RC (Fig. 3c). This implies that as one DOX resides in the PBP, the DBP is unlikely to bind another DOX. In another word, the two binding pockets of the binding protomer are unlikely to accommodate two DOXs simultaneously.

It is worth noting that conformational changes can also be observed in the other two protomers, though the changes are less evident than those in the binding protomer (Figure S4, Supporting Information). For example, as the PC1-PC2 distance in the binding protomer declined remarkably in the PBP region, the distance in the access protomer increased moderately, while no obvious change was observed in the extrusion protomer (Figure S4a, Supporting Information). In terms of the gyration radius of DBP, the change in the binding protomer apparently exhibits negative correlation with that in the other two protomers around the PBP region (Figure S4b, Supporting Information). Therefore the observations are in general agreement with previous studies, which suggest conformational coupling among AcrB protomers during substrate transport^13^,^22^,^23^,^34^.

### The switch-loop causes a 3 kcal/mol barrier between the binding pockets

The energy barrier separating PBP and DBP is relatively low (~3 kcal/mol) (Fig. 2a). As indicated by various AcrB/substrate complex structures and mutagenesis studies, the barrier is mainly caused by the switch-loop lying between the binding pockets, which constricts the translocation pathway and possesses an evident influence on the efflux rate and the substrate specificity^3^,^14^,^18^,^19^. Further inspection shows that the switch-loop exhibits higher backbone root-mean-squared fluctuations as DOX passes through (Figure S5; Supporting Information). The enhanced flexibility of the loop may facilitate barrier crossing of the substrate. Moreover, the relatively small molecular size of DOX may also contribute to lower the barrier as the steric clash between substrate and AcrB is largely avoided. Due to the low energy barrier, the drug molecule can easily transit between the discrete binding pockets.

## Discussion

RND family transporter AcrB is a principal multidrug exporter, which has been studied most extensively as a prototype of similar pumps. Although previous experimental evidences have established the framework of working mechanism of AcrB, many details of interactions and processes that functionally govern and regulate the efficacy of the RND pump system remain elusive. Therefore, obtaining any affinity, kinetic and dynamic parameters could be useful for the rational design of new antibacterial agents for the therapy of multidrug resistance infections. In this study, we obtained the difference between the binding free energy of doxorubicin to the two substrate binding pockets, PBP and DBP, as well as the energy barrier separating them. These values are evaluated for the first time at the level of all-atom simulations with all the inter-atomic energy terms treated rigorously. Most importantly, it was found that DOX binds to the PBP with similar affinity as that to the DBP. The substrate binding affinity of PBP has been evaluated for cephalothin (relative molecular mass, *M*_r_ ~ 396) and erythromycin (*M*_r_ ~ 734) using the molecular mechanics-generalized Born surface area (MM-GBSA) approach^16^. The MM-GBSA calculation shows that the smaller (or low-molecular-mass) substrate binds at the DBP 6.5 kcal/mol more stable than at the PBP, whereas the larger (or high-molecular-mass) one is 10.9 kcal/mol less stable at the DBP, suggesting that one of the binding sites would predominate during the translocation of a specific substrate^6^,^17^. This notion, however, is challenged by our simulations of doxorubicin (*M*_r_ ~ 544). In another words, substrate binding to AcrB is not solely determined by a single binding site as proposed previously^35^. It is reasonable that the relative binding affinities of DBP and PBP may vary among the structurally and chemically different substrates, thereby affecting the apparent substrate binding affinity and the efflux rate of AcrB. Another functional implication of the finding is that the PBP may also play a role in the inhibitory mechanism of the efflux pump inhibitors (EPI). Some EPIs have been shown to inhibit substrate efflux by competing with the substrates for the binding sites inside the DBP^11^,^35^. In the cases that the substrate binding at the PBP significantly affects the efflux efficiency, the EPI might interfere the substrate binding at PBP.

The conformational change analyses based on ABF simulations revealed a stable intermediate state between the access and binding states with a closed PC1/PC2 cleft and a substrate bound in the PBP. This intermediate state provides new clues for understanding substrate binding and selectivity. The conformational flexibility of the PC1/PC2 cleft was identified in the previous crystallographic and simulation studies of AcrB^22^,^26^,^36^. Here, we found that substrate binding at PBP will induce the closure of the cleft and form a stable conformational state. PC cleft closure before the substrate binding in the DBP will obviously prevent the substrate back diffusion. At the same time, it will also prevent the influx of non-substrates, providing a possible control for substrate selectivity. Since crystal structures show that the PC cleft is widely open in the binding protomer, it was proposed that the PC cleft tunnel functions as an exit for non-substrate to leave the porter domain^36^. Our simulation results suggest that this is unlikely. Rather, the cleft functions as a flexible lid, and presumably opens the entrance to uptake substrates from periplasm and shields it to prevent back diffusion of substrates and influx of non-substrates.

To sum up, our work provides the missing link in the translocation mechanism of AcrB by showing that substrate can form stable binding in the PBP of binding protomer. The binding also accompanies significant conformational changes, giving an intermediate state between the access and binding states. The findings entail a more detailed stepwise mechanism for substrate translocation (Fig. 4). The translocation process starts with the attachment of substrate to the lateral PC1/PC2 cleft in the access protomer (Fig. 4a,d). During the transformation from the access state to the binding state, the substrate achieves optimal binding inside the PBP with high affinity, and the PC1/PC2 cleft closes to prevent backflow of substrate (Fig. 4b,e). Transition to the DBP is readily to occur with a relatively low energy barrier. As the substrate forms stable binding in the DBP, the PC1/PC2 cleft re-opens, giving the binding state as observed in many asymmetric AcrB structures (Fig. 4c,f). This stepwise process of substrate translocation conforms the peristaltic pump mechanism, showing that the conformational flexibility of the porter domain is prerequisite for substrate binding and transportation, and the functional rotating involves more intermediate states other than the access, binding and extrusion states (Fig. 4g). It is, however, also worth noting that substrates with different properties than DOX could give very different free energy profiles of translocation^24^ and require different kinds of conformational changes during the functional rotation, which are all interesting topics in the future studies.

## Methods

### System Setup

The initial complex structure of AcrB and doxorubicin (DOX) was derived from a combination of two crystal structures (2DR6^4^ and 2GIF^7^) as reported by R. Schulz and A. Vargiu *et al*.^20^,^21^,^28^ The former structure is a drug bound form at 3.3 Å, but some loops such as residue 499 to 512 are missing, whereas the latter is a more complete structure with higher resolution (2.9 Å) but with no substrate. After superimposing the two structures, the DOX in the distal binding pocket (DBP) of 2DR6 and the transporter of 2GIF were combined and used as the initial structure.

The relative orientation of AcrB with respect to the lipid membrane was predicted through the PPM server^37^. Then the AcrB-DOX complex was embedded into a pre-equilibrated 1-palmitoyl-2-oleoyl-sn-glycero-3-phosphocholine (POPC) lipid bilayer following an in-house script modified from inflateGRO^38^. The central cavity among the TM domains was filled with 18 POPC molecules (9 in the upper leaflet and 9 in the lower leaflet) to avoid proton leakage across membrane^5^,^39^. The system was subsequently immersed in a periodic box of approximately 140 × 140 × 170 Å^3^, containing 320,520 atoms, and neutralized by Na^**+**^ ions. Standard protonation states were adopted for ionizable residues.

### Conventional Molecular Dynamics Simulation

All the simulations reported here were performed with the parallel molecular dynamics package NAMD 2.8^40^ using CHARMM27 force fields for protein and lipids^41^,^42^,^43^, and TIP3P model^44^ for water. The parameters for doxorubicin were derived through the program CGenFF (v0.9.1 beta)^45^ with the CGenFF force field (v2b6)^46^,^47^. The amino group in the daunosamine moiety of DOX is protonated according to the pKa inquired from DrugBank^32^. Short-range non-bonded interactions were calculated using a switching distance of 10 Å and a cutoff at 12 Å. The long-range electrostatic interactions were evaluated via particle-mesh Ewald (PME) method^48^, with a grid spacing of 1 Å. Covalent bonds involving hydrogen atoms were constrained by the SHAKE algorithm^49^, allowing a time step of 2 fs. In the NVT simulations, the temperature was kept at 300 K using the Langevin thermostat, and for the NPT simulations, the pressure was maintained at 1.01325 bar using the Nosé-Hoover Langevin piston pressure control^50^. The system was equilibrated using the following sequence of steps: (i) 5000-step energy minimization with protein backbone and doxorubicin restrained; (ii) 500-ps NVT simulation with protein, doxorubicin and lipids fixed; (iii) 200-ps semi-isotropic NPT simulation with protein, doxorubicin and lipids fixed; (iv) 200-ps isotropic NPT simulation with only protein and doxorubicin fixed, followed by 500-ps semi-isotropic NPT simulation; (v) 500-ps NVT simulation with protein backbone and doxorubicin restrained, (vi) 600-ps NPT simulation with gradually decreasing restraints on protein backbone and doxorubicin. Finally, a 50-ns production run was carried out in semi-isotropic NPT ensemble without any restraint. The final structure was used as the initial structure of the adaptive basing force simulation.

### Adaptive biasing force simulation

The adaptive basing force (ABF) method^29^,^30^, implemented in the *COLVARS* module of NAMD 2.8^40^, was utilized to delineate the free energy profile of DOX translocation. This method couples the formalisms of thermodynamic integration and average force with unconstrained molecular dynamics, and has been widely applied to the studies on chemical and biological problems^29^,^30^. In the framework of ABF, an intuitive reaction coordinate (RC) 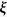 is defined, then the free energy is constructed from its derivative:


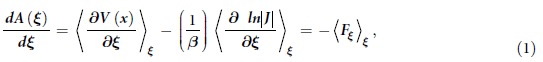


where 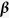 equals to 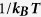 (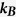 is the Boltzmann constant and 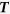 is the temperature), 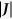 is the Jacobian determinant for the transformation of generalized to Cartesian coordinates, and  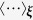  denotes the ensemble average at 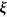. The first term of the ensemble average in (1) accounts for physical force acting on the system, derived from the potential function 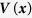, and the second term is a pure geometric correction. In practice, the instantaneous force 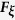 is accumulated in small bins with finite size 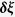, producing the mean force 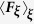 from the running ensemble average. In the ABF simulations, a real-time biasing force 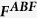 is applied to counteract the mean force, allowing the system to overcome existing barriers along 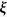 and leading to a more uniform sampling on the predefined RC:


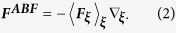


**Figure 1:**
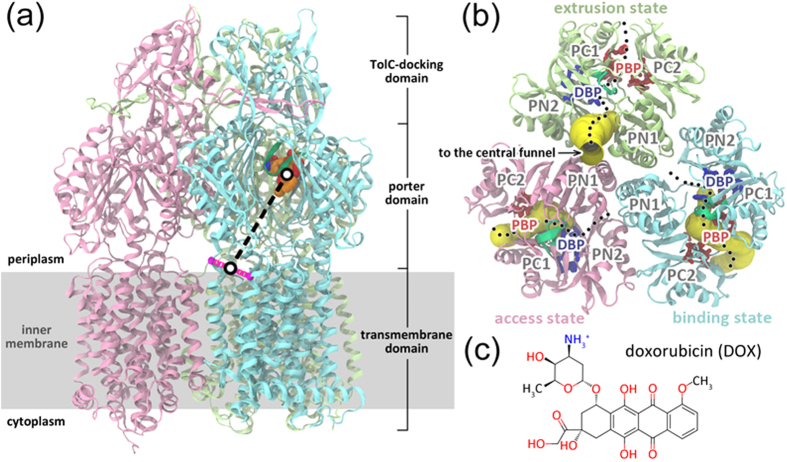
The simulation system. (**a**) Ribbon diagram of the asymmetric structure of AcrB trimer. DOX bound in the DBP of binding protomer is shown in van der Waals representation (orange, blue and red for carbon, nitrogen and oxygen atoms, respectively). The reaction coordinate (black dashed line) is defined as the distance between the midpoint (black circle) of the C_α_ atoms of residues Val557 and Asn871 (magenta spheres connected by magenta dotted line) and the mass center of DOX (black circle). (**b**) Top view of the porter domains looking down from the periplasm. The residues lining the PBP and DBP are depicted in stick model and colored in red and blue, respectively. The switch-loop is highlighted in dark green. The translocation pathway within each porter domain is outlined by black dots, and the cavity along each pathway is represented by yellow tube. The cavities are defined with the protein analysis and visualization software CAVER^53^. (**c**) Molecular structure of doxorubicin.

**Figure 2:**
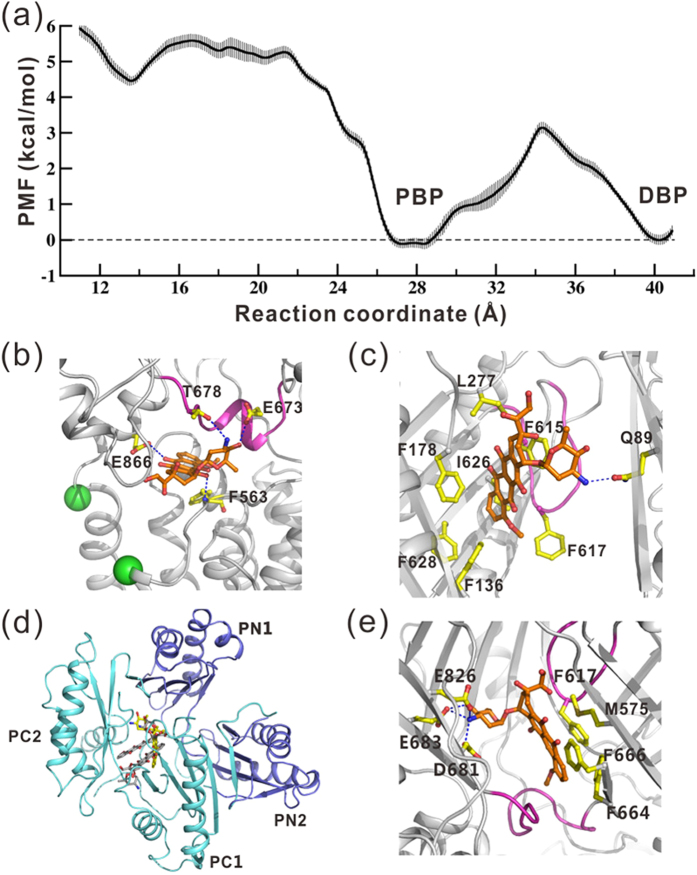
Energetics of DOX translocation in the binding protomer. (**a**) The PMF profile of DOX translocation along the reaction coordinate with error bars. The two deep energy minima correspond to DOX binding at the PBP and DBP. (**b**) A representative snapshot of DOX bound near the entrance corresponding to the shallow energy minimum at RC = 13.6 Å. DOX (orange for carbon atoms) and the residues (yellow for carbon atoms) interacting with it are drawn as a ball-stick model. Their oxygen and nitrogen atoms are colored in red and blue, respectively. The hydrogen bonds are represented by blue dashed lines and the PC-loop is highlighted in magenta. The C_α_ atoms of residues Val557 and Asn871 are represented by green spheres. (**c**) A representative snapshot of DOX bound in the DBP. The switch-loop is highlighted in magenta. (**d**) Top view of DOX (yellow for carbon atoms) bound in the PBP. The DOX dimer (grey for carbon atoms) bound at the PC1/PC2 cleft in the crystal structure (PDB ID: 4DX7) is shown by superimposing the access protomer of 4DX7 with the binding protomer of the snapshot in (**e**). (**e**) A representative snapshot of DOX bound in the PBP (RC = 26.5 ~ 28.5 Å). The PC-loop and the switch-loop are highlighted in magenta.

**Figure 3:**
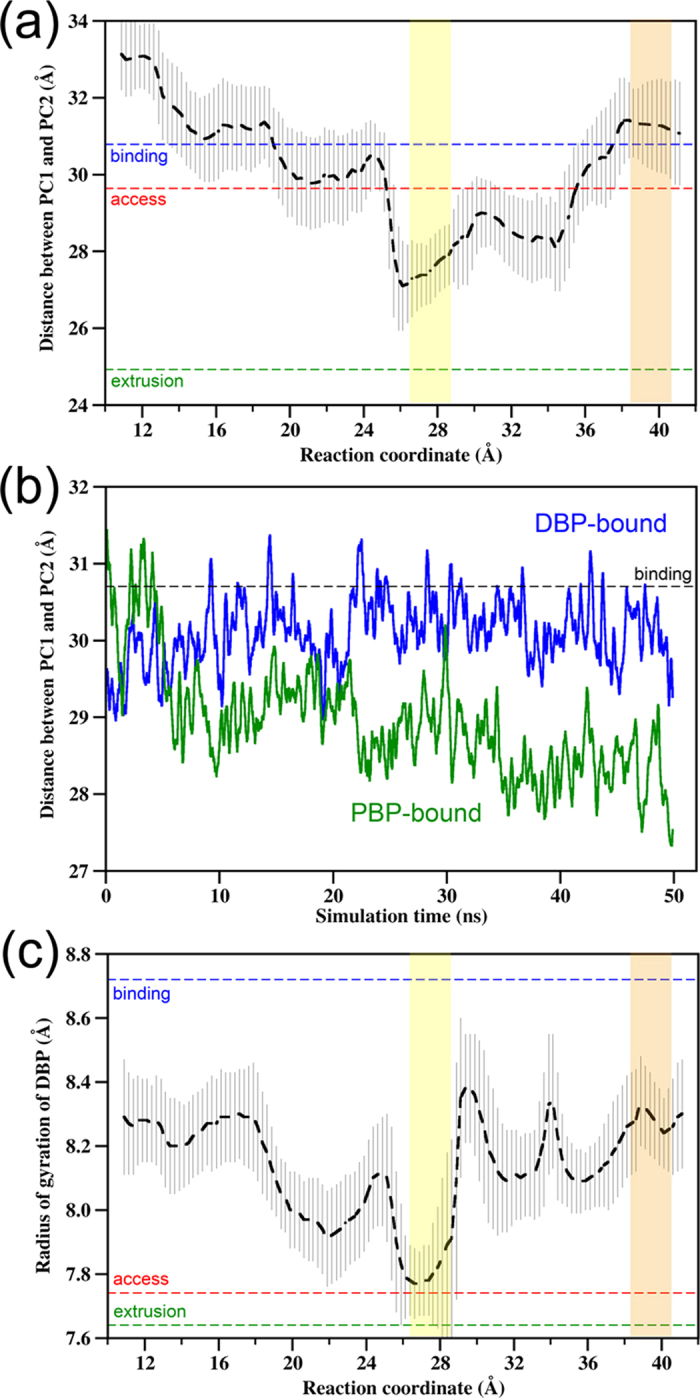
Conformational changes upon DOX binding in the PBP. (**a**) Variation of the average distance between the centers of mass of subdomains PC1 and PC2 along the RC with error bars. The PC1-PC2 distances in the crystal structure are indicated by the horizontal dashed lines. The PBP and DBP regions are highlighted with the yellow and orange shaded bands, respectively. (**b**) Time evolution of the distance between the centers of mass of PC1 and PC2 subdomains with DOX bound in the PBP (green) or in the DBP (blue) in the 50-ns unbiased trajectories. (**c**) Variation of the average radius of gyration of the DBP against the RC with error bars.

**Figure 4:**
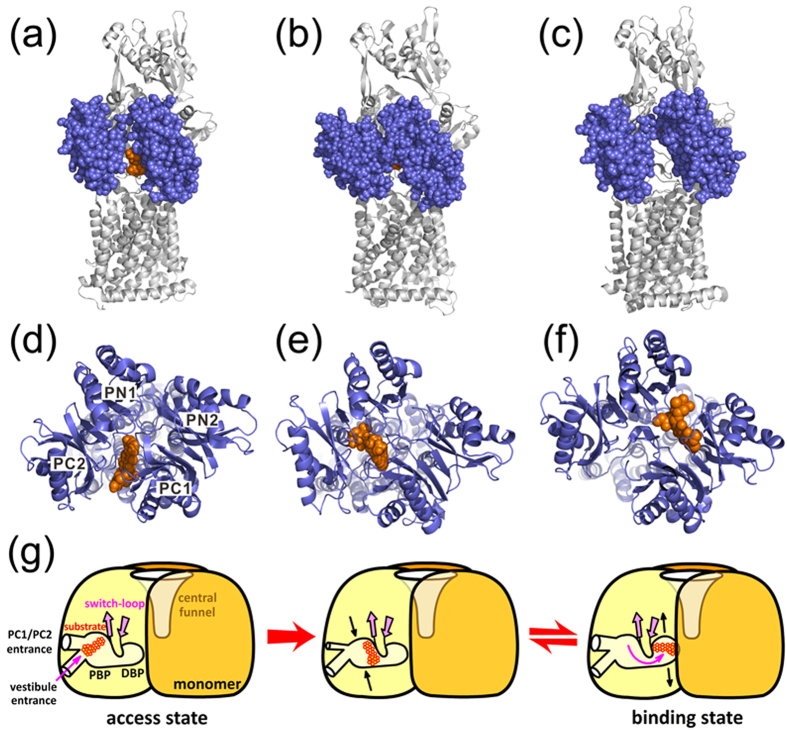
Stepwise substrate translocation during the access to binding transition in the functional rotating mechanism of AcrB. (**a**,**d**) Side and top views of the access protomer with DOX bound at the lateral PC1/PC2 cleft in the crystal structure 4DX7 (with one of the DOXs removed). DOX is represented by VDW mode and colored in orange. In the side view, the PC1 and PC1 subdomains are shown in VDW model and colored in ice blue. (**b**,**e**) Side and top views of the binding protomer with DOX bound in the PBP observed in the ABF simulations. (**c**,**f)** Side and top views of the binding protomer with DOX bound in the DBP in the crystal structure 4DX7. (**g**) A cartoon diagram showing substrate translocation and conformational changes during the access to binding transition of AcrB.

For the AcrB system, the RC 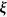 was defined as the distance between the substrate center of mass (CoM) and the midpoint of the line connecting the 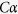 atoms of Val557 (in TM7 helix) and Asn871 (in TM8 helix) (Fig. 1a). The zero-point of 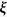 is situated near the TM8/TM9 groove which is proposed to be the vestibule entrance^6^,^7^,^8^,^9^,^24^,^31^. The distal binding pocket (DBP) is located at about 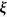 = 39 Å, and the proximal binding pocket (PBP) at about 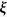 = 28 Å. For a better characterization of the translocation process, we defined the sampling range of 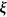 from 11 to 41 Å. For the regions with 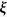 < 11 Å, the substrate has extensive interactions with the phospholipids, and suffers a severe convergence problem. To enhance the efficiency of sampling, the sampling range was divided into 7 non-overlapping windows, and sampled separately. The windows are 5.0 Å in width, except the first and the last ones which are 2.5 Å each. The instantaneous force exerted along 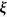 was accrued in 0.1-Å-sized bins, where the average mean force was evaluated. To avoid nonequilibrium effects in the dynamics of the system, initial 1000 samples in each bin were accumulated for the evaluation of average mean force prior to the application of biasing force. The initial structures in the seven windows were selected from a full-window ABF simulation across the entire reaction coordinate. For most windows, the simulation time lasted for 150 ns to guarantee the convergence of ABF simulation. The sampling procedure produced at least 400,000 samples in each bin, and the total simulation time added up to 1.01 μs.

The coarse upper-bound limit of the standard error (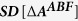) in the derived free energy difference (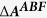) between points 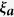 and 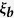 can be estimated using the following formulation^30^,^51^:


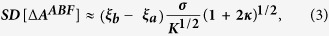


where 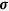 denotes the standard deviation of thermodynamic forces along 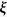, 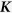 is the total number of force samples, and 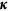 is the correlation length for the series of computed forces, which was evaluated based on an analysis of the autocorrelation as described in ref^52^.

## Additional Information

**How to cite this article**: Zuo, Z. *et al*. Stepwise substrate translocation mechanism revealed by free energy calculations of doxorubicin in the multidrug transporter AcrB. *Sci. Rep*. **5**, 13905; doi: 10.1038/srep13905 (2015).

## Supplementary Material

Supplementary Information
