# Supplementary material for: Stepwise substrate translocation mechanism revealed by free energy calculations of doxorubicin in the multidrug transporter AcrB
Source: Sci Rep. 2015 Sep 14;5:13905. doi: 10.1038/srep13905 (PMC4595977; doi:10.1038/srep13905)
Supplement: Supplementary Information [file srep13905-s1.pdf]

SUPPORTING INFORMATION

Stepwise substrate translocation mechanism revealed by free energy calculations of doxorubicin in the multidrug transporter AcrB

Zhicheng Zuo, Jingwei Weng*, Wenning Wang*

Shanghai Key Laboratory of Molecular Catalysis and Innovative Materials, Department of Chemistry, and ‡Institutes of Biomedical Sciences, Fudan University, Shanghai, P.R. China

***Email: wnwang@fudan.edu.cn**

***Email: jwweng@fudan.edu.cn**

Supplemental Figures

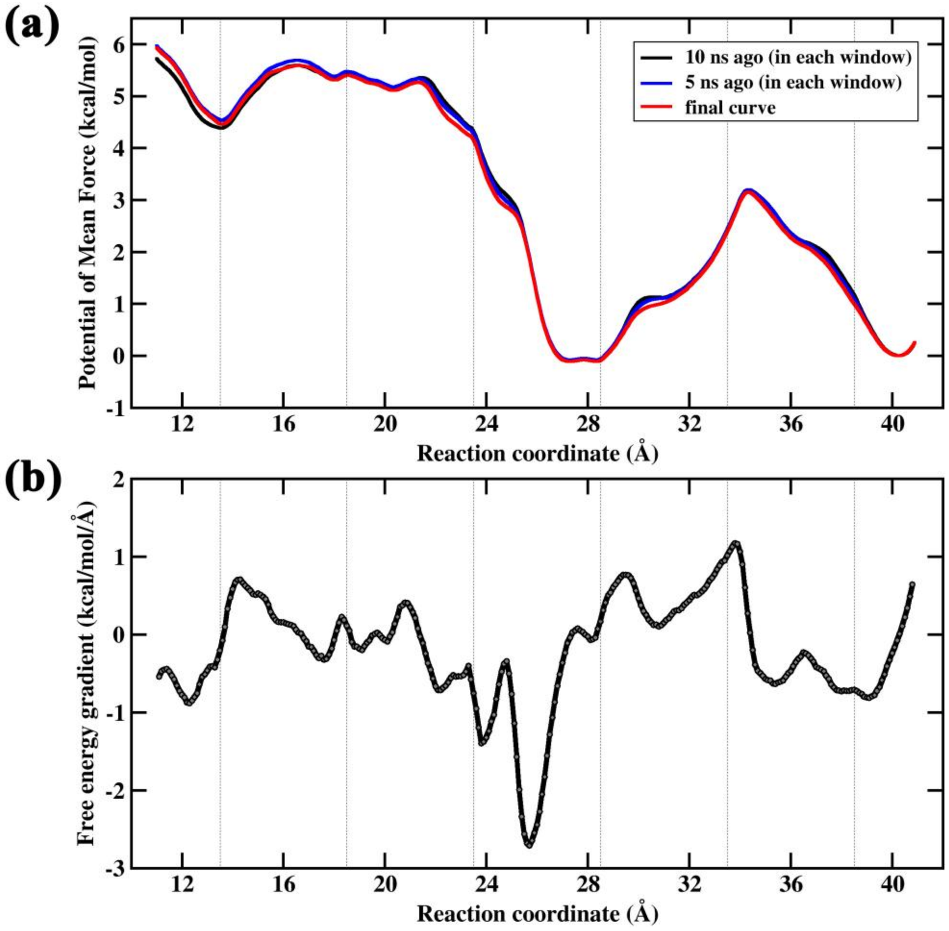

Figure S1. Convergence of the ABF simulation. (a) The evolution of the free energy profile in the last 10 ns of the ABF simulation (in each window). (b) The first derivative of free energy (i.e. energy gradient) at the end of ABF simulation.

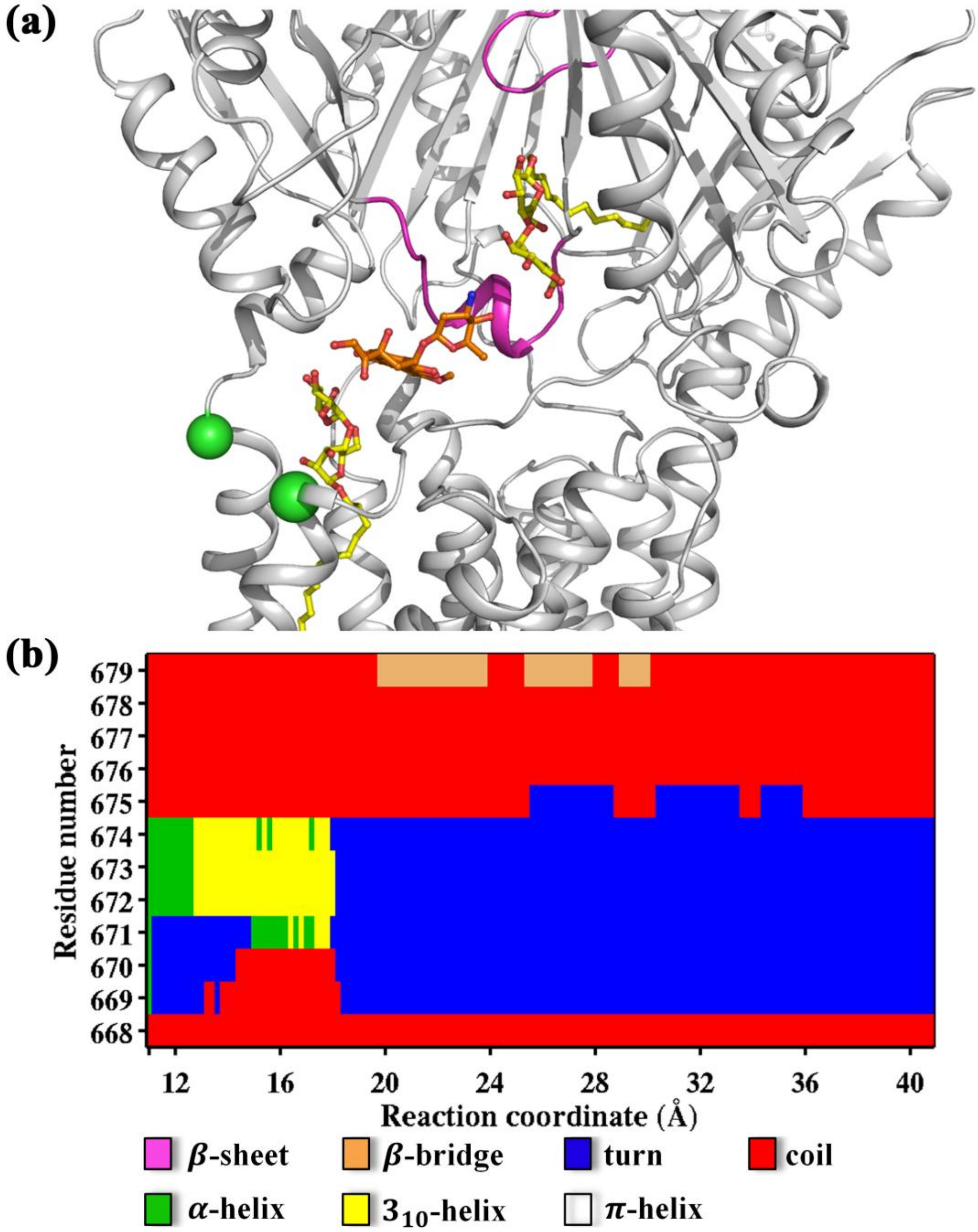

Figure S2. (a) The location of DOX (stick mode, orange for carbon atoms) in the local energy minimum at RC=13.6 Å lying between the two detergent molecules (stick mode, yellow for carbon atoms) in the crystal structure. For DOX and the detergent molecules, the oxygen atoms are colored in red and the nitrogen atom is colored in blue. The C_α atoms of residues Val557 and Asn871 are represented by green spheres. The switch-loop and PC-loop are colored in magenta. (b) The variation of the secondary structure of the PC-loop along the RC.

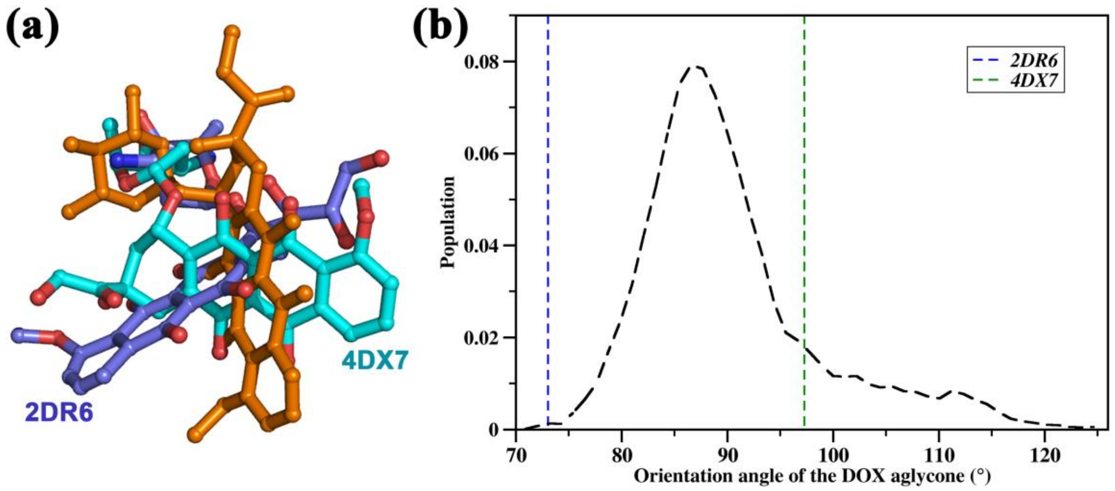

Figure S3. (a) Orientations of DOX in the DBP shown in the crystal structures (cyan and violet) and in the ABF simulation (orange). (b) Distribution of the orientation angle of DOX when the substrate stays inside the DBP (RC = 38.5 ~ 40.5 Å). The orientation angle of DOX is defined as the angle formed between the aglycone moiety and the Nβ6 strand (residue 173 to 178) in the PN2 subdomain of AcrB. The orientation angles observed in the crystal structures are denoted by vertical dashed lines.

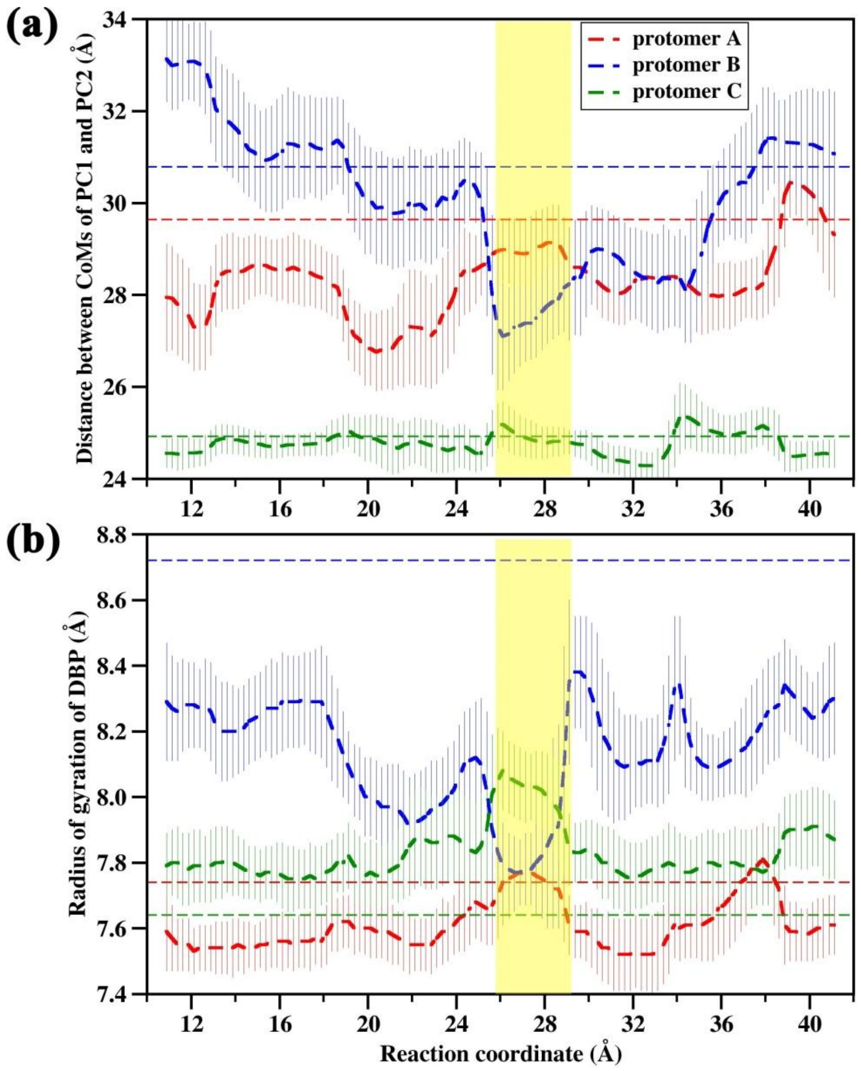

Figure S4. Conformational coupling among protomers upon DOX binding in the PBP. (a) Variation of the distance between the centers of mass (CoM) of subdomains PC1 and PC2 against the RC. (b) Variation of the radius of gyration of DBP against the RC. The red, blue and green dashed curves are plotted for protomers A, B and E in the ABF simulations, respectively, and the dashed horizontal lines denote the corresponding values in the crystal structure. The PBP region is highlighted by the yellow shaded bands.

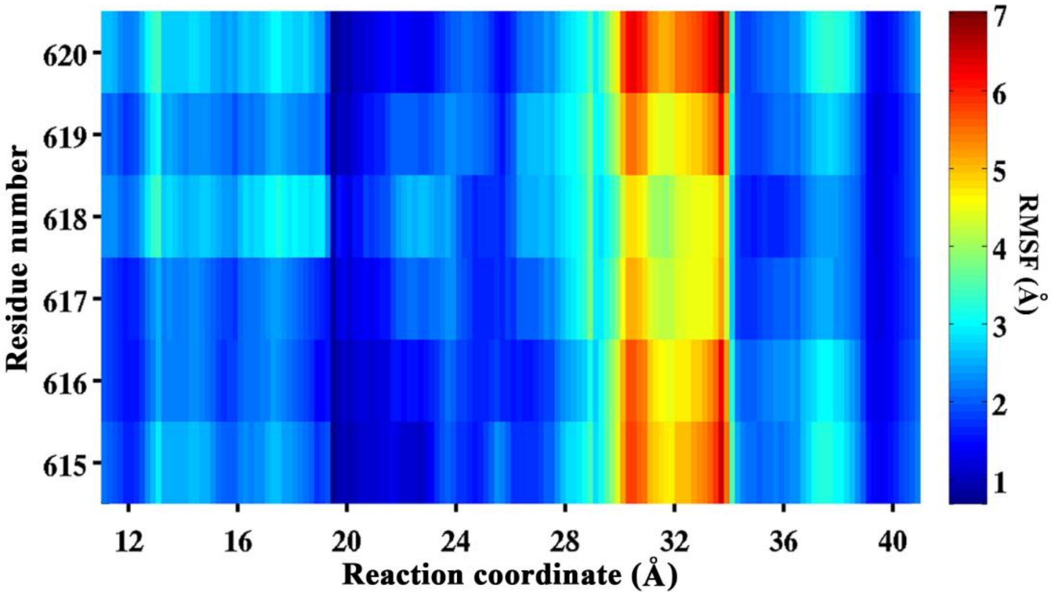

Figure S5. Variation of the backbone root-mean-squared fluctuations (RMSFs) of the switch-loop residues along the RC. The color bar denotes scale of the RMSF values.

Supplemental Tables

Table S1. Interactions between DOX and the residues lining the DBP with occurrence more than 20% in the simulation (RC = 38.5 ~ 40.5 Å) and those observed in the crystal structures.

Interaction patterns	Residues	Simulations (occurrence %)	2DR6[c]	4DX7[d]
Hydrogen bond[a]	Gln89	47	√	√
	Gly179	22	√	
	Phe136	32		√
	Phe178	69	√	√
Van der Waals interaction[b]	Ile277	55	√	√
	Phe615	100	√	√
	Phe617	62	√	
	Ile626	69		
	Phe628	76		

[a] Hydrogen bond is defined as the distance between the donor and accepter atoms is below 3.5 Å and the angle formed by the donor, hydrogen, and acceptor atoms is less than 35° from 180°.

[b] Van der Waals interaction is defined as the separation between any carbon atom in DOX and any carbon atom on the hydrophobic side chain of AcrB is below 4.0 Å.

[c] Appearance or not of the interactions in the crystal structure 2DR6[1].

[d] Appearance or not of the interactions in the crystal structure 4DX7[2].

Table S2. Interactions between DOX and the PBP with occurrence more than 20% in the simulation (RC = 26.5 ~ 28.5 Å) in comparison with those observed in the crystal structures or identified by biochemical characterizations.

interaction patterns	residues	simulations (occurrence %)	4DX7 proximal-PC1[d]	4DX7 proximal-PC2[e]	fluorescence assays[f]	mutagenesis[g]
hydrogen bond[a]	Phe617	38			√	
	Asp681	81		√		
	Glu683	30				
	Asn719	7		√		
	Glu826	95				
salt-bridge[b]	Asp681	90		√		
	Glu683	70				
	Glu826	98		√		
van der Waals interaction[c]	Met575	41	√	√		
	Phe617	91	√	√	√	
	Phe664	21	√		√	√
	Phe666	92	√		√	√
	Leu668	40	√		√	
	Val672	29				
	Leu828	17		√		
	Met862	< 5		√		

[a] Hydrogen bond interaction is defined as the distance between the donor and accepter atoms is below 3.5 Å and the angle formed by the donor, hydrogen, and acceptor atoms is less than 35° from 180°.

[b] Salt-bridge interaction is defined as the distance between the nitrogen atom in the daunosamine moiety of DOX and the carboxylic oxygen atoms of acidic residues (Glu and Asp) on AcrB is below 4.0 Å.

[c] Van der Waals interaction is defined as the separation between any carbon atom in DOX and any carbon atom on the hydrophobic side chain of AcrB is below 4.0 Å.

[d] Interactions observed in the crystal structure (PDB ID: 4DX7) between the proximal-PC1 DOX and the PBP of the access monomer.

[e] Interactions observed in the crystal structure (PDB ID: 4DX7) between the proximal-PC2 DOX and the PBP of the access monomer.

[f] Residues labeled by a lipophilic dye-maleimide (also an AcrB substrate) as

quantified by fluorescence assays[3].

[g] Mutagenesis in the amino acid residue which decreases the efflux activity[4-6].

Supplemental references

1. Murakami, S., Nakashima, R., Yamashita, E., Matsumoto, T. & Yamaguchi, A. Crystal structures of a multidrug transporter reveal a functionally rotating mechanism. *Nature* **443**, 173-179 (2006).
2. Eicher, T. *et al.* Transport of drugs by the multidrug transporter AcrB involves an access and a deep binding pocket that are separated by a switch-loop. *Proc. Natl. Acad. Sci. U. S. A.* **109**, 5687-5692 (2012).
3. Husain, F. & Nikaido, H. Substrate path in the AcrB multidrug efflux pump of Escherichia coli. *Mol. Microbiol.* **78**, 320-330 (2010).
4. Yu, E. W., Aires, J. R., McDermott, G. & Nikaido, H. A periplasmic drug-binding site of the AcrB multidrug efflux pump: a crystallographic and site-directed mutagenesis study. *J. Bacteriol.* **187**, 6804-6815 (2005).
5. Nakashima, R., Sakurai, K., Yamasaki, S., Nishino, K. & Yamaguchi, A. Structures of the multidrug exporter AcrB reveal a proximal multisite drug-binding pocket. *Nature* **480**, 565-569 (2011).
6. Takatsuka, Y. & Nikaido, H. Site-directed disulfide cross-linking shows that cleft flexibility in the periplasmic domain is needed for the multidrug efflux pump AcrB of Escherichia coli. *J. Bacteriol.* **189**, 8677-8684 (2007).
